# Association between self-rated health and physical performance in middle-aged and older women from Northeast Brazil

**DOI:** 10.7717/peerj.8876

**Published:** 2020-04-10

**Authors:** Sabrina Gabrielle Gomes Fernandes, Catherine M. Pirkle, Tetine Sentell, José Vilton Costa, Alvaro Campos Cavalcanti Maciel, Saionara Maria Aires da Câmara

**Affiliations:** 1 Postgraduate Program in Rehabilitation Sciences, Faculty of Health Sciences of Trairi, Federal University of Rio Grande do Norte, Santa Cruz, Rio Grande do Norte, Brazil; 2 Office of Public Health Studies, University of Hawaii at Manoa, Honolulu, HI, USA; 3 Department of Demography and Actuarial Sciences, Federal University of Rio Grande do Norte, Natal, Rio Grande do Norte, Brazil; 4 Department of Physiotherapy, Federal University of Rio Grande do Norte, Natal, Rio Grande do Norte, Brazil

**Keywords:** Health, Self-report, Muscle strength, Aging, Women

## Abstract

**Background:**

Self-rated Health (SRH) is regarded as a simple and valid measure of a person’s health status, given its association to adverse health outcomes, including low physical performance in older populations. However, studies investigating these associations in low- and middle-income settings are scarce, especially for middle-aged populations. Understanding the validity of SRH in relation to objective health measures in low-income populations could assist in decision making about health policy and strategies, especially in under-resourced settings.

**Objective:**

Assess the relationship between SRH and physical performance measures in middle-aged and older women in a low-income setting of Brazil.

**Methods:**

This is a cross-sectional study of 571 middle-aged (40–59 years old) and older (60–80 years old) women living in Parnamirim and Santa Cruz in the Northeast region of Brazil. Participants reported their health status and were allocated to the “SRH good” or “SRH poor” groups. The physical performance evaluation included: handgrip strength, one-legged balance with eyes open and closed and chair stand test. The relationship between SRH and physical performance for middle-aged and older women was assessed by quantile regression (modeling medians) adjusted for potential confounders (age, socioeconomic variables, body mass index, menopause status, age at first birth, parity, chronic conditions and physical activity).

**Results:**

Middle-aged women from the “SRH good” group presented better physical performance with 1.75 kgf stronger handgrip strength (95% CI [0.47–3.02]; *p* = 0.004), 1.31 s longer balance with eyes closed ([0.00–2.61]; *p* = 0.030), and they were 0.56 s faster in the chair stand test ([0.18–0.94]; *p* = 0.009) than those who reported “SRH poor”. No association was found for balance with eyes open. For older women, there was no evidence of associations between physical performance and SRH.

**Conclusion:**

This study showed that SRH is significantly associated with objective measures of physical performance in a sample of low-income middle-aged women. SRH can be an important tool to indicate the need for further evaluation of physical performance among middle-aged women and can be particularly useful for low-income communities.

## Introduction

Self-rated health (SRH) is one of the most commonly utilized outcome measures in social epidemiology, public health research and clinical practice (Cesari et al., 2008). It might better capture the burden of clinical and subclinical conditions compared to the traditionally adopted measures of disease, such as scales for measuring decline in physical and cognitive functioning and direct measures of blood pressure or heart rate (Cesari et al., 2008), because it includes a wider spectrum of information than many clinical tests can capture. SRH has been associated with morbidity and mortality in a variety of populations, including older men and women from various countries (Idler & Benyamini, 1997; Assari, 2016; Han et al., 2005).

Self-rated health is a simple and inexpensive measure that places lower demands on the assessor and allows evaluation even from a distance (Wang et al., 2018), which can be advantageous for low-income and rural communities. On the other hand, it may be influenced by sociocultural background and education and some individuals may consider different constructs of health with it, such as a specific health condition rather than their overall health (Krause & Jay, 1994).

Physical performance measures, such as grip strength, balance and chair stands, are determined by physiological functions that typically decline with age and are considered valuable tools for identifying early stages of functional limitation (Guralnik et al., 1995). These physical performance tests have proven valid and reliable and can predict important outcomes such as institutionalization, mortality, and disability in diverse populations (Cesari et al., 2008). These are all outcomes of growing public health concern as populations age at an accelerated rate globally. Evaluating the association of SRH with objective measures of physical performance may provide information that helps to guide preventive actions to reduce the rates of adverse health outcomes in older populations.

Some evidence shows that SRH is associated with physical performance in older populations (Pagotto, Bachion & Silveira, 2013; Pérez-Zepeda et al., 2016); however, to our knowledge, studies investigating this association in middle-aged women are rare (Kanagae et al., 2006). Yet physical function impairments related to aging appear to initiate early in midlife, particularly for women with low socioeconomic status (Murray et al., 2011). During this phase of life, important changes occur in women. For example, muscle mass tends to decrease gradually after the third decade of age and declines at an accelerated rate after the fifth decade, a time in life that correlates with the onset of menopause (Silva et al., 2016). If middle-aged women reporting poor health status also present worse physical function, the evaluation of SRH may help to identify earlier those with higher risk of subsequent disability. Such early identification of at-risk women could prove useful for targeted prevention interventions, particularly in low-income and remote settings, given the fact that SRH is a simple and inexpensive assessment tool and can be useful in achieving greater population coverage in areas with insufficient health services.

Studies investigating the association of SRH and physical function at any age in low- and middle-income settings, such as Brazil, are also rare (Pérez-Zepeda et al., 2016; Cullati et al., 2018) and conclusions from studies with high-income populations may not be applicable to lower-income settings. Some authors have noted that individuals from high-income locations, where there are more educational opportunities and health facilities, are in a better position to evaluate their own health status than individuals from low-income settings, where they may be less aware of adverse health conditions affecting them (Sen, 2002; Huisman, Van Lenthe & Mackenbach, 2007). Qualitative research suggests possible differences in health perception between socioeconomic groups, demonstrating the multidimensionality of people’s view of health. People from higher-income settings seem to consider varied dimensions when assessing their health, including elements of being fit, active and the absence of illness, whereas those from lower-income settings seem to limit themselves more to functional aspects (Benyamini et al., 2003).

Given that little research has been conducted on SRH in Brazil, more investigation into this subject is needed, especially in regions such as the Northeast where the number of health-related research projects is low compared to the more developed regions of the country (Silva et al., 2015). There has been one exception, Pérez-Zepeda et al. (2016). Employing data from older adults from four countries, including two in Latin America (Brazil and Colombia), the authors concluded that the prevalence of poor physical function increased as perceptions of health worsened for all research sites. They did not include middle-aged adults in their analyses.

Understanding the validity of SRH in relation to objective health measures in low-income populations could help in decision making about health policy, especially in communities where diagnostic resources are less available, such as in low- and middle-income countries.

Thus, the purpose of this study is to assess the relationship between SRH and physical function, in middle-aged and older women from a low-income setting of Brazil. Brazil, especially the Northeast region, is an important place to conduct this work due to the high social inequalities that affect individuals’ access to quality health care. We hypothesize that middle-aged and older women who report poor SRH will have worse physical performance, as previous studies document a graded association between both SRH and physical performance. Correlating physical performance scores with SRH can provide insights on the utility of SRH in a population of middle-aged and older women from a poor region in Brazil, with relevance to other low-income settings.

## Methods

This study was conducted using data collected in Parnamirim and Santa Cruz, two cities in the Rio Grande do Norte state of Northeast Brazil. Parnamirim is located in the metropolitan region of Natal, the capital of Rio Grande do Norte, and has approximately 202,456 inhabitants. Santa Cruz is located in the countryside, 122 km from Natal and has approximately 39,660 inhabitants. This article presents a secondary analysis of data from a research study that aimed to examine the influence of menopause and hormone levels on sarcopenia and physical function (Azevedo et al., 2018; Câmara et al., 2015a). Data were collected between 2014 and 2016.

### Population and sample

The study population was composed of women aged between 40 and 80 years living in Parnamirim and Santa Cruz. A convenience sample was obtained by advertisements in all primary care centers across both cities. The advertisements included basic information about the study objectives, procedures and inclusion criteria and information on how to contact the research team. The exclusion criteria of the primary study included the following: neurological disease, such as Parkinson’s, stroke or any condition compromising evaluation of physical function measures; and four or more errors on the Leganès Cognitive Test (LCT), a validated screening tool used to identify cognitive impairment (Caldas et al., 2012), which is considered indicative of the inability to complete the study procedures. Of the 589 evaluated women, 18 were excluded because they were not able to classify their SRH, leading to a final sample size of 571 women.

### Procedures

All women were assessed by trained interviewers (physiotherapists or physiotherapy students) in a community center in Parnamirim and at the Federal University of Rio Grande do Norte in Santa Cruz. The standardized protocols used for this assessment are described below.

### Physical performance

Physical performance was assessed with four tests: handgrip strength, one-legged balance eyes open and closed, and chair stand test.

- *Handgrip strength:* Grip strength is a test used to assess global muscle strength and a criterion to identify sarcopenia and frailty among older populations (Clegg et al., 2013; Cruz-Jentof et al., 2010; Fried et al., 2001). Grip strength is quantified by measuring the amount of static force that the dominant hand can squeeze around a Saehan^®^ dynamometer. Participants were positioned as recommended by The American Society of Hand Therapist (Fess, 1992), seated, with their elbow by their side and flexed to a right angle and a neutral or slightly extended (up to 30 degrees) wrist position. Participants were asked to squeeze the dynamometer with maximal isometric effort for five seconds (Câmara et al., 2015a). The test was performed three times with 1-min intervals between them (Amaral et al., 2019). Intraclass correlation coefficients (two-way mixed effects, absolute agreement, single measurement) showed that the three attempts were highly correlated (ICC = 0.883; 95% CI [0.848–0.908]; *p* < 0.001). The mean of the three trials was used in analyses (Silva et al., 2016; Câmara et al., 2015a, 2015b).
- *Balance test*: This test is used to identify whether a balance problem exists and to determine its underlying cause (Mancine & Horak, 2010). To assess balance, researchers asked participants to stand on a single leg, without any help, up to a maximum of 30 s. The duration for which the participants could maintain this position was timed for each leg using a stopwatch. The test was performed first with participants’ eyes open and then with eyes closed for each leg, for a total of four tests (Cheng et al., 2009). Measurements were recorded as the mean values (in seconds) of trials with the eyes open and closed (Cheng et al., 2009). The ICC (two-way mixed effects, absolute agreement, single measurement) for the two attempts with eyes open (ICC = 0.692; 95% CI [0.646–0.732]; *p* < 0.001) and eyes closed (ICC = 0.524; 95% CI [0.461–0.581]; *p* < 0.001) showed that the measures were moderately correlated, which is expected since the balance performance for each leg varies according to the participant’s laterality. Using mean values, we observed the participant’s general balance performance.
- *Chair stands:* The chair stand is one of the most important clinical tests for lower limb function evaluation and is reflective of the most demanding daily life activities (e.g., climbing stairs, getting out of a chair or bath tub, rising from a horizontal position) (Jones, Rikli & Beam, 1999). Participants were asked to stand up and sit down five times consecutively as quickly as possible (Guralnik et al., 1995). The time required to complete the five repetitions was recorded in seconds and used for analyses.

### Self-rated health

Self-rated health was collected using a single question “Would you say your health in general is: excellent, very good, good, fair or poor?” For the analyses, we categorized the answers into two groups: good health (excellent, very good and good), and poor health (fair and poor) (Kanagae et al., 2006; Meireles et al., 2015). Given the relatively small sample size in this study, we dichotomized SRH since the small numbers in some categories would lead to less precise estimates for them.

#### Potential confounders

##### Age

This variable was included because the frequency of health problems differs among age groups and because younger people may interpret information about their own health differently from older people (Jylha, 2009). Moreover, older people tend to present worse physical performance (Câmara et al., 2015a). The participant’s age was collected through self-reporting during the initial evaluation. Participants were divided into two age groups: 40 to 59 years (middle-aged group) and 60 to 80 years (older adult group).

##### Socioeconomic variables

Previous research shows that low education and low income are strongly associated with poor perceived health (Szwarcwald et al., 2005). Moreover, lower socioeconomic status has been associated with lower physical function (Murray et al., 2011). The variables of family income and education were self-reported. Family income was categorized using the Brazilian minimum monthly wage as a reference (MW), which is the minimum amount that employers can legally pay workers in Brazil. In our study, it was dichotomized as less than 3 MW and 3 MW or more (Câmara et al., 2015a). Education was assessed by the number of years that the subject attended school and then dichotomized as less than basic education (<8 years) and basic education or more (≥8 years).

##### Body Mass Index (BMI)

BMI was considered a potential confounder since it is associated with SRH (Hunger & Major, 2015) and to physical performance (Câmara et al., 2015a) in previous studies. BMI was calculated from measured height (m), measured by a Welmy^®^ W100H scale with stadiometer and weight, measured by the Wiso^®^ W930 scale. Participants were evaluated without shoes and wearing light clothing. They were asked to stand in an upright position with their vision line parallel with the Frankfort plane. To measure height, participants were asked to stand with their feet together in a parallel position and to remove any clips or other items from their hair. Height was taken from the tallest point of the participant’s head. BMI was categorized according to the World Health Organization’s international classification: 18.5 to 24.99 (normal weight); 25.00 to 29.99 (Overweight); ≥30.00 (Obese).

##### Physical activity (PA)

Participating in PA can result in improvements in overall health and reduce risks associated with a sedentary lifestyle (Bailis, Segall & Chipperfield, 2003). PA has been widely studied in relation to SRH, with the general finding that moderate and/or frequent leisure PA is associated with higher SRH (Bailis, Segall & Chipperfield, 2003). This variable was measured by asking participants if they were currently taking part in sports, exercise, or other PA in their leisure time at least three times per week and for 30 min or more each time (U.S. Department of Health & Human Services, 2018). This variable was dichotomized as yes or no.

##### Chronic conditions

Studies examining the association between SRH and chronic diseases such as diabetes mellitus (Badawi et al., 2012), chronic lung disease (Nguyen, Cuenco & Corrieri-Kohlman, 2008), high blood pressure (Carlsson, Andreasson & Wandell, 2011), arthritis (Uutela et al., 2016), depression (Ambresin et al., 2014) and cancer (Roelsgaard et al., 2016) are common (Mavaddat et al., 2014). There is an association between chronic conditions—such as cancer (Dimeo et al., 1997), diabetes mellitus (Astrom et al., 2018), and depressive symptoms (Gomes et al., 2013)—and physical functioning. Participants were asked if a doctor or nurse had told them they had any of the following: diabetes, chronic lung disease, high blood pressure, coronary heart disease, arthritis, depression and cancer. For the analyses we condensed the responses into: 0–2 chronic conditions and 3 or more chronic conditions.

##### Menopause status

Menopause is a critical event that occurs at midlife for women. Hormonal changes related to menopause have been associated with diminished health status (Dennerstein, Dudley & Guthrie, 2003). Some research on menopause documents an increase in the presence of irritating symptoms that adversely affect women’s self-perception of health (Dennerstein, Dudley & Guthrie, 2003). Also, several studies (Câmara et al., 2015a; Cooper et al., 2008) have found associations between menopause and poorer physical performance. In our study, menopause status was determined using the Stages of Reproductive Aging Workshop classification—STRAW (Harlow et al., 2012). Women were classified into three groups: *premenopausal* (those reporting regular menses), *perimenopausal* (those reporting irregular menses, with differences on cycle length over 7 days or amenorrhea up to 1 year) *and postmenopausal* (those reporting absence of menses for over 1 year). Women who reported having a hysterectomy were included in the postmenopausal group.

##### Age at first birth and parity

These variables were considered potential confounders since previous research has found associations between multiparity and early age at first birth with worse physical function (Câmara et al., 2015a; Pirkle et al., 2014). Furthermore, these variables are also associated with several health conditions that may occur years after giving birth, including increased cardiovascular risk and chronic conditions (Pirkle et al., 2014) that may affect self-perception of health. For this study, age at first birth and parity were self-reported and categorized according to previous studies (Câmara et al., 2015a, 2015b). Age at first birth was divided in two groups: those reporting the first birth before 18 years; those reporting the first birth at 18 years of age or older and those who had never given birth (nulliparous). Parity was dichotomized as 0–2 childbirths and 3 or more childbirths.

### Ethics

All participants were informed of the objectives and procedures of the research study and signed a consent form. The study protocol was approved by the Ethics and Research Committee of Federal University of Rio Grande do Norte (approval number: 1.875.802).

### Data analysis

Analyses were conducted using Statistical Package for Social Sciences (SPSS 20.0) and The R Project for Statistical Computing version 3.6.1 (R Core Team, 2019). First, descriptive statistics for all variables were calculated by SRH groups and the variables were compared using Chi-square tests.

Physical performance variables were calculated by independent variable categories and compared with a Mann–Whitney test or Kruskal–Wallis test with post-hoc Dunn’s test. Additionally, we calculated median differences with 95% CIs for all physical performance measures according to SRH.

Multiple quantile regression (QR) analyses (modeling medians) were conducted to observe the association between SRH and each of the physical performance measures, adjusted for covariates. This approach is a more robust estimation method than Ordinary Least Squares regression (OLS) since it is not sensitive to violations of assumptions such as heteroscedasticity, error non-normality, and outliers. QR coefficients are interpreted similarly to those of OLS coefficients except that a QR coefficient indicates the changes in the conditional quantile as a parametric function of the explanatory variables change in the value at the modeled percentile and not the conditional mean, of the dependent variable (Koenker, 2005). First, stratified analyses were performed by age group. For each physical performance measure, four models were performed, according to groups of covariates to evaluate the effect of each group in the association and the potential for some of the variables to be on the causal pathway. The first model presents the unadjusted results. In the second model, we included the socioeconomic variables (age, education and family income). In the third model, we added variables related to reproductive history (menopausal status, parity and age at first childbirth). In the fourth model, we added variables of physical fitness (BMI and PA) and chronic conditions. Finally, to test possible interaction between SRH and age group for each measure of physical performance, an interaction term between these two variables was added to the four previously adjusted regression models. The models were estimated using R software, from the linear quantile function “rq ()” of the “quantreg” package (version 5.35) (Koenker et al., 2018).

**Figure 1:**
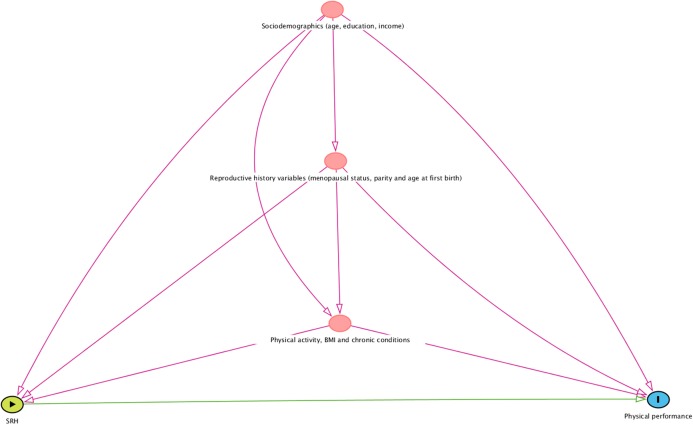
A visual representation of the conceptual framework informing the statistical analyses. SRH indicates self-rated health; BMI, body mass index. The figure was built using the Dagitty software (http://www.dagitty.net/dags.html#).

Figure 1 shows a visual representation of the conceptual framework underpinning our analyses. We used the approach of increasing adjustment by groups of variables to be able to assess how each block of variables influences the results and the potential for some of the variables to be on the causal pathway.

## Results

The sample characteristics according to SRH are presented in Table 1. Although the sample was recruited using a convenience sampling strategy, the socioeconomic characteristics are similar to other community-based studies in the area (Gomes et al., 2013; Maciel & Guerra, 2007) as well as to the entire population of women from Parnamirim and Santa Cruz according to the most recent census data (Brazilian Institute of Geography and Statistics (IBGE), 2012).

**Table 1:** Sample characteristics (*N* = 571).

Variables	Self-rated health		
	Good *N* (%)	Poor *N* (%)	*p* value^a^
Total	178 (31.2%)	393 (68.8%)	
Age categories			0.040
Middle-age (40–59 years)	143 (33.5%)	284 (66.5%)	
Older adults (60–80 years)	35 (24.3%)	109 (75.7%)	
Education*			0.002
Basic education or more (≥8 years)	113 (36.8%)	194 (63.2%)	
Less than basic education (<8 years)	65 (24.8%)	197 (75.2%)	
Family income**			0.007
≥3 Minimum wages	67 (39.0%)	105 (61.0%)	
<3 Minimum wages	110 (27.6%)	288 (72.4%)	
BMI (Kg/m^2^)			0.315
Normal	40 (37.0%)	68 (63.0%)	
Overweight	76 (30.6%)	172 (69.4%)	
Obese	62 (28.8%)	153 (71.2%)	
Physical activity			0.016
Yes	79 (37.3%)	133 (62.7%)	
No	99 (27.6%)	260 (72.4%)	
Chronic conditions*			<0.001
0–2	169 (34.0%)	328 (66.0%)	
3 or more	9 (12.5%)	63 (87.5%)	
Menopausal status^§^			0.062
Premenopausal	42 (35.0%)	78 (65.0%)	
Perimenopausal	32 (39.5%)	49 (60.5%)	
Postmenopausal	100 (27.6%)	262 (72.4%)	
Age at first birth			0.088
≥18 years-old or nulliparous	150 (32.8%)	307 (67.2%)	
<18 years-old	28 (24.6%)	86 (75.4%)	
Parity			0.015
0–2	89 (36.6%)	154 (63.4%)	
3 or more	89 (27.1%)	239 (72.9%)	

**Notes:**

* 2 missing values.

** 1 missing value.

§ 8 missing values.

a *p* value for Chi-Square test.

BMI, Body Mass Index.

The groups of SRH were statistically different in relation to age, education, family income, PA, chronic conditions and parity. A significantly higher proportion of older women (75.7%) reported their health as “poor” compared with middle-aged women (66.5%) (*p* = 0.040). Moreover, a higher proportion of women with lower education (75.2% vs. 63.2%; *p* = 0.002), lower family income (72.4% vs. 61.0%; *p* = 0.007), more chronic conditions (87.5% vs. 66.0%; *p* < 0.001), less PA (72.4% vs. 62.7%; *p* = 0.016) and having had more children (72.9% vs. 63.4%; *p* = 0.015) reported their health as poor compared to the other groups. No statistically significant associations were observed in relation to SRH and BMI, menopausal status, and age at first birth.

Table 2 presents the medians and the 25th and 75th percentiles (Q25–Q75) of the physical performance tests according to SRH and covariates. Considering the total sample, women reporting “good health” presented better physical performance on all the tests; that is, they had greater handgrip strength (27.50 kgf [23.33–30.00] vs. 25.33 kgf [21.50–28.67]; *p* < 0.001), longer balance times with eyes open (26.90 s [16.95–30.00] vs. 23.26 s [11.34–30.00]; *p* = 0.002) and with eyes closed (5.73 s [3.77–10.66] vs. 4.61 s [2.80–8.15]; *p* < 0.001), and performed the chair stand test faster (9.60 s [8.52–10.74] vs. 10.37 s [9.00–12.25]; *p* < 0.001). Similarly, women with higher educational levels had better performance for all tests. Compared to obese women, those with a normal weight according to BMI showed better results for both balance tests (eyes open and closed) and for the chair stand test. Postmenopausal women performed more poorly in all tests compared to premenopausal women. They also had a weaker handgrip strength and shorter balance time with eyes open than perimenopausal women. Perimenopausal women performed more poorly on the balance test with eyes closed than premenopausal women. In general, women with more chronic conditions and those with increased parity presented worse results than the others.

**Table 2:** Median levels of physical performance according to covariates (*N* = 571).

	Handgrip strength (kgf)	One-legged balance-eyes open (s)*	One-legged balance-eyes closed (s)*	Chair stands (s)*
**SRH**	**Median [Q25–Q75]**			
Good	27.50 [23.33–30.00]	26.90 [16.95–30.00]	5.73 [3.77–10.66]	9.60 [8.52–10.74]
Poor	25.33 [21.50–28.67]	23.26 [11.34–30.00]	4.61 [2.80–8.15]	10.37 [9.00–12.25]
*p* Value^a^	<0.001	0.002	<0.001	<0.001
Education				
Basic education or more (≥8 years)	26.67 [23.33–30.00]	27.04 [17.08–30.00]	5.62 [3.37–10.21]	9.93 [8.50–11.35]
Less than basic education (<8 years)	25.00 [21.33–28.67]	19.72 [8.77–30.00]	4.37 [2.72–7.92]	10.33 [9.10–12.59]
*p* Value^a^	<0.001	<0.001	<0.001	0.001
Family income				
≥3 minimum wages	27.17 [23.33–30.00]	24.82 [12.81–30.00]	5.05 [2.95–9.29]	10.07 [8.53–11.40]
<3 minimum wages	25.50 [22.00–29.00]	24.53 [12.85–30.00]	5.01 [2.99–8.89]	10.19 [8.90–11.88]
*p* Value^a^	0.010	0.329	0.808	0.162
BMI				
Normal	25.00 [21.42–28.67]	28.13 [18.69–30.00]	6.16 [3.37–11.88]	9.81 [8.48–11.10]
Overweight	26.00 [22.67–29.33]	25.47 [12.55–30.00]	5.05 [2.95–9.38]	10.11 [8.79–11.84]
Obese	26.33 [22.00–30.00]	21.36 [10.86–30.00]	4.59 [2.82–7.41]	10.33 [9.15–11.91]
*p* Value^b^	0.105	0.002^c^	0.014^c^	0.036^c^
Physical activity				
Yes	27.00 [22.67–29.33]	25.65 [14.38–30.00]	5.34 [3.37–9.53]	10.09 [8.78–11.48]
No	25.33 [22.00–29.33]	23.48 [11.58–30.00]	4.70 [2.73–8.75]	10.13 [8.82–11.91]
*p* Value^a^	0.104	0.246	0.045	0.551
Chronic conditions				
0–2	26.00 [22.33–29.33]	25.53 [13.47–30.00]	5.20 [3.11–9.68]	10.06 [8.73–11.66]
3 or more	25.00 [21.33–28.00]	17.23 [7.45–24.87]	3.66 [2.43–5.82]	10.36 [9.39–12.32]
*p* Value^a^	0.062	<0.001	<0.001	0.078
Menopausal Status				
Premenopausal	27.00 [23.67–30.67]	28.14 [19.13–30.00]	6.51 [3.78–12.35]	9.66 [8.49–10.77]
Perimenopausal	27.67 [23.17–30.83]	26.89 [18.90–30.00]	5.37 [2.97–8.89]	9.87 [8.51–11.28]
Postmenopausal	25.33 [21.33–28.67]	21.36 [10.13–30.00]	4.55 [2.65–8.20]	10.34 [9.00–12.24]
*p* Value^b^	<0.001^d, e^	<0.001^d, e^	<0.001^d, f^	<0.001^d^
Age at first child				
≥18 years-old or nulliparous	26.00 [22.33–29.33]	24.89 [12.97–30.00]	5.05 [2.97–9.73]	10.13 [8.79–11.73]
<18 years-old	25.33 [21.83–29.00]	24.50 [12.72–30.00]	4.94 [2.80–8.08]	10.08 [8.77–11.90]
*p* Value ^b^	0.055	0.710	0.590	0.895
Parity				
0–2	26.67 [22.67–29.33]	27.04 [16.40–30.00]	5.67 [3.15–10.96]	9.94 [8.60–11.29]
3 or more	25.33 [22.00–29.33]	21.92 [10.50–30.00]	4.63 [2.84–8.01]	10.25 [8.97–12.22]
*p* Value^a^	0.116	<0.001	0.002	0.011

**Notes:**

* 2 missing values.

a *p* value for Mann–Whitney test.

b *p* value for Kruskal–Wallis test.

c Obese ≠ normal.

d postmenopausal ≠ premenopausal.

e postmenopausal ≠ perimenopausal.

f perimenopausal ≠ premenppausal.

SRH, Self-rated health; BMI, Body Mass Index; Q25–Q75, 25th and 75th percentiles.

Higher values indicate better performance for grip strength, and the balance tests, and worse performance for chair stands.

Table 3 shows differences of physical performance tests according to SRH, stratified by age group. When dividing the sample according to age group, SRH remained associated with the physical performance measures only in the middle-aged group, where those reporting good health status presented significantly better performance in all tests (handgrip strength: 28.33 kgf [24.00–30.67] vs. 26.00 kgf [22.33–29.33], *p* < 0.001; balance eyes open: 29.93 s [20.21–30.00] vs. 25.90 s [16.03–30.00], *p* = 0.008; balance eyes closed: 6.97 s [4.10–12.14] vs. 5.26 s [3.16–9.26], *p* = 0.002; chair stands: 9.47 s [8.42–10.42] vs. 10.05 s [9.25–11.90], *p* = 0.003). For the older group, those reporting good SRH performed the chair stands faster than those reporting poor SRH (10.31 s [9.25–11.90] vs. 11.78 s [10.15–13.77], *p* = 0.012). No significant differences were observed for all the other physical performance tests according to SRH in the older adults’ group (handgrip strength: 23.67 kgf [20.67–27.67] vs. 23.33 kgf [20.33–26.83], *p* = 0.485; balance eyes open: 11.63 s [7.36–25.56] vs. 12.26 s [4.53–24.84], *p* = 0.463; balance eyes closed: 4.03 s [2.49–5.05] vs. 3.11 s [1.87–5.35], *p* = 0.204).

**Table 3:** Differences of physical performance tests according to SRH, stratified by age group (middle-aged and older women) (*N* = 571).

	Handgrip strength (kgf)	One-legged balance-eyes open (s)*	One-legged balance-eyes closed (s)*	Chair stands (s)*
	**Median [Q25–Q75]**			
**SRH**	**Middle-aged group, 40–59 years-old (*N* = 427)**			
Good	28.33 [24.00–30.67]	29.93 [20.21–30.00]	6.97 [4.10–12.14]	9.47 [8.42–10.42]
Poor	26.00 [22.33–29.33]	25.90 [16.03–30.00]	5.26 [3.16–9.26]	10.05 [9.25–11.90]
*p* Value^a^	<0.001	0.008	0.002	0.003
Median difference (95% CI)	2.33 [1.00–3.33]	4.03 [−0.09 to 5.83]	1.71 [0.26–3.60]	4.03 [−0.01 to 5.75]
**SRH**	**Older adults’ group, 60–80 years-old (*N* = 144)**			
Good	23.67 [20.67–27.67]	11.63 [7.36–25.56]	4.03 [2.49–5.05]	10.31 [9.25–11.90]
Poor	23.33 [20.33–26.83]	12.26 [4.53–24.84]	3.11 [1.87–5.35]	11.78 [10.15–13.77]
*p* Value^a^	0.485	0.463	0.204	0.012
Median difference (95% CI)	0.33 [−1.67 to 3.00]	−0.63 [−5.86 to 8.86]	0.92 [−0.46 to 1.70]	−1.47 [−2.68 to −0.18]

**Notes:**

* 2 missing values.

a *p* value for Mann–Whitney test.

SRH, Self-rated health; Q25–Q75, 25th and 75th percentiles.

Table 4 shows the quantile regression results for physical performance variables according to SRH for the middle-aged and older groups. For the middle-aged group, those reporting “good health” had 1.75 kgf (95% CI [0.47–3.02]; *p* = 0.004) stronger grip strength, sustained 1.31 s ([0.00–2.61]; *p* = 0.030) longer in the balance test with eyes closed, and were 0.56 s ([0.18–0.94]; *p* = 0.009) faster in the chair stand test compared to the “poor health” group, even in the fully adjusted model. For the balance test with eyes open, there was no association with SRH in the fully adjusted model (“good health” group with 0.81 s more; [−0.94 to 2.57]; *p* = 0.290). For the older adults’ group, no statistically significant differences were found for the physical performance measures in relation to SRH (“good health” group had a weaker handgrip strength of 0.13 kgf ([−2.02 to 1.76]; *p* = 0.654), stayed in the balance test with eyes open 1.41 s less ([−4.71 to 1.89]; *p* = 0.581), stayed in the balance test with eyes closed 0.54 s more ([−0.34 to 1.41]; *p* = 0.756) and were 0.72 s faster in the chair stand test ([−1.75 to 0.31]; *p* = 0.172).

**Table 4:** Quantile regression models for physical performance measures according to self-rated health (good vs. poor), stratified by age categories (poor SRH is the reference category) (*N* = 571).

	Model 1	Model 2	Model 3	Model 4
	**b (95% CI)**			
	**Middle-aged group (40–59 years-old)**			
Handgrip strength (kgf)	2.28 [1.16–3.41]	1.65 [0.43–2.88]	2.22 [0.87–3.56]	1.75 [0.47–3.02]
*p* Value	0.001	0.013	0.004	0.004
One legged balance-eyes open (s)^(§)^	3.64 [1.34–5.94]	1.37 [−0.14 to 2.88]	2.02 [0.64–3.40]	0.81 [−0.94 to 2.57]
*p* Value	0.001	0.033	0.006	0.290
One legged balance-eyes closed (s)^(§)^	1.40 [−0.20 to 3.00]	1.64 [0.35–2.93]	1.73 [0.58–2.89]	1.31 [0.00–2.61]
*p* Value	0.037	0.027	0.015	0.030
Chair Stands (s)^(§)^	−0.39 [−0.83 to 0.06]	−0.52 [−0.96 to −0.09]	−0.56 [−0.99 to −0.13]	−0.56 [−0.94 to −0.18]
*p* Value	0.008	0.010	0.064	0.009
	**Older adults’ group (60–80 years-old)**			
Handgrip strength (kgf)	0.75 [−1.41 to 2.91]	−0.35 [−2.30 to 1.61]	0.60 [−1.18 to 2.37]	−0.13 [−2.02 to 1.76]
*p* Value	0.449	0.831	0.801	0.654
One legged balance-eyes open (s)^(§)^	−0.47 [−7.47 to 6.53]	−1.31 [−6.90 to 4.29]	−1.22 [−5.67 to 3.22]	−1.41 [−4.71 to 1.89]
*p* Value	0.896	0.929	0.495	0.581
One legged balance-eyes closed (s)^(§)^	1.06 [−0.02 to 2.15]	0.99 [0.25–1.73]	0.41 [−0.61 to 1.43]	0.54 [−0.34 to 1.41]
*p* Value	0.070	0.228	0.481	0.756
Chair stands (s)^(§)^	−1.17 [−2.35 to 0.00]	−0.67 [−1.91 to 0.56]	−0.62 [−1.31 to 0.08]	−0.72 [−1.75 to 0.31]
*p* Value	0.009	0.131	0.024	0.172

**Notes:**

§ 2 missing values.

SRH, Self-rated health.

Model 1, Unadjusted; Model 2, Adjusted for age, education and family income; Model 3, Adjusted for age, education, family income, parity, age first birth and menopausal status; Model 4, Adjusted for age, education, family income, parity, age first birth, menopausal status, body mass index, physical activity and chronic conditions.

Higher values indicate better performance for grip strength and the balance tests, and worse performance for chair stands.

When assessing the interaction between SRH and age group adjusted for the potential confounders, the results were not statistically significant for any physical performance measures in the fully adjusted model: handgrip strength (1.82 kgf; 95% CI [−0.67 to 4.31]; *p* = 0.284), balance with eyes open (1.50 s; [−5.83 to 8.84]; *p* = 0.779), balance with eyes closed (0.94 s; [−0.45 to 2.34]; *p* = 0.351) and chair stands (0.96 s; [−0.29 to 2.22]; *p* = 0.097) (Supplemental File). There was only one statistically significant result for the interaction between SRH and age group in model 3 (adjusted for age, education, family income, parity, age first birth and menopausal status) for the chair stand test (1.57 s; [0.29–2.85], *p* = 0.041). While there were relatively large coefficients for the interaction term for the grip strength and one legged, open-eyed balance tests, the confidence interval crossed zero and increasing adjustment for potential confounders reduced the strength of the association.

## Discussion

This study investigated the association between SRH and objective measures of physical performance in a sample of low-income middle-aged and older women from Northeast Brazil. The results showed that women reporting good health status had, overall, better physical performance than those reporting poor health status. However, when analyzing middle-aged and older women separately, the differences between the means of physical performance tests according to SRH were statistically significant only for the middle-aged sample. There was no significant association between SRH and physical performance among the older women sample in the adjusted models. However, when examining effect interaction between the dichotomous SRH and age group variables, the results were not statistically significant, possibly given our relatively small sample size and categorization of these variables.

When compared to previous studies that investigated the association between SRH and objective measures of physical function, our findings present some divergences. While we were not able to show that SRH is associated with physical performance in the older group, several previous studies have showed significant associations using different measures of physical function in older populations (Bez & Neri, 2014; Belmonte et al., 2017). Bez & Neri (2014), investigating a sample of 689 older men and women from Southeast Brazil (one of the most developed regions of this country), found that those in the lowest quartile for gait speed had a significantly higher likelihood of reporting poor health. Similar to our findings for the older group, they did not observe an association between SRH and grip strength. In a study conducted by Belmonte et al. (2017), 2,558 elderly male and female individuals over 65 years of age from all regions of Brazil (the FIBRA study) answered questionnaires about functional capacity (basic and instrumental daily life activities) and performed handgrip strength and gait speed tests. Those reporting poor SRH were more dependent in their daily living activities, had slower gait speed and had weaker handgrip strength than those reporting good health status. The authors did not analyze the associations according to study site, which limits the comparison of their results with our study, given our lower-income setting.

Pérez-Zepeda et al. (2016) analyzed data from 1,995 older adults (65–74 years) who participated in the International Mobility in Aging Study (IMIAS), which included samples from very distinct socioeconomic settings including Natal in Northeast Brazil. In this study, the authors found significant associations between SRH and physical functioning. More specifically, they found that older women from Natal reporting poor SRH had 2.6 higher odds of having poor physical performance in the Short Physical Performance Battery (SPPB). Although the SPPB includes lower-limb physical performance tests similar to ours, such as chair stands and balance, the results are interpreted through a composite score that represents the participants’ performance on the three lower limb tests. This may limit comparison with our results, since we examined the association between SRH and each of the tests rather than generating a composite score for all three together. Additionally, grip strength was not included in the SPPB used by the other authors.

Some hypotheses may explain the lack of association between SRH and objective measures of physical performance for older women in our sample. It is possible that low-income older women participating in this study consider other aspects of their life apart from physical function when evaluating their health status. Previous studies have shown that low-income older people, despite presenting worse physical function (Murray et al., 2011) and worse health status in general compared to their high-income peers (Sen, 2002), tend to seek fewer health services and have lower adherence to treatments (Pagotto, Bachion & Silveira, 2013). This can be due, among other factors, to their undervaluation of less limiting conditions. Objective measures of physical performance, like those we used in this study, are useful in identifying early signs of physical impairment related to aging even among high functioning individuals (Cesari et al., 2008). Although small reductions in the means of the physical performance tests are considered clinically relevant (Perera et al., 2014), we believe that low-income older people might consider these small reductions in physical performance as “normal” signs of aging, rather than signs of poor health. Cultural aspects associated with lower levels of education and income and lower expectations regarding the health status of the elderly may interfere with their health assessment (Pagotto, Bachion & Silveira, 2013). It has also been reported that older people living in low-income regions may overestimate their health in order to show self-sufficiency, for fear of institutionalization or showing that they are in need of assistive care (Carvalho et al., 2012).

The association between SRH and physical performance measures in middle-aged populations has been less investigated in the literature. As far as we know, only the study by Kanagae et al. (2006), which was conducted in Japan, evaluated the association between SRH and physical performance in a middle-aged sample of women. Similar to our findings, the authors found that women reporting poor health presented weaker grip strength compared to those reporting good health.

Middle-age is marked by menopause, an important milestone of a woman’s life. Menopause is associated with reductions in means of physical performance tests such as grip strength (Câmara et al., 2015a). This reduction in physical performance can affect individuals’ social roles, since during this phase, women must adapt to psycho-affective and socio-cultural changes that may have repercussions on health status and quality of life (Silva et al., 2016). Those symptoms can discourage women and reduce their overall activities, resulting in poorer physical performance and their overall health assessment, which may explain the significant association between SRH and all physical performance measures for this group.

The physical performance measures investigated in these analyses are well-known important predictors of adverse health-related outcomes in older populations, including elevated risk of falls, earlier mortality, institutionalization and hospitalization (Fogel et al., 2000; Guralnik et al., 1995; Inouye et al., 1998; Studenski et al., 2003). The possibility of being able to identify those at higher risk for poor health outcomes in an earlier stage of the life-course, such as the midlife, could have long term implications for optimizing the aging process (Lachman, 2015). Being able to sooner identify these warning signs for poor health status may inform strategies and policies targeted at delaying, minimizing, or preventing some of the adverse outcomes that typically occur in later life (Lachman, 2015).

### Limitations

The cross-sectional design of this study does not allow us to extend the results to affirm that poorer physical performance is causally associated with negative health perception or vice-versa. Longitudinal studies are necessary to elucidate the direction of this relationship. Moreover, the relatively small sample size did not allow us to present results for each level of SRH, rather than dichotomizing it as we did, since this would have reduced the power of our analyses. Additionally, although we included objective measures of physical performance, some of the confounding variables we included such as PA and chronic conditions were collected by self-report, which may have led to misclassification. By stratifying the analyses by age group and categorizing continuous confounders such as education and BMI into categorical variables, there may still be residual confounding. Finally, future research may want to explore measuring SRH on a continuous scale, rather than as a single item ordinal measure, in order to improve the power of analyses. For studies conducted among middle-aged women, collecting data on menopause-associated physical and mental changes, and discomforts, may be useful in better understanding the observed associations, especially for interpreting the results in this specific age group.

## Conclusion

The results showed that SRH is associated with objective measures of physical performance in a sample of low-income middle-aged women. Thus, middle-aged women reporting poor health status should be considered for a more complete physical performance evaluation using objective measures in order to identify those at high risk of adverse outcomes. The lack of association between SRH and physical performance for the older women shows that the benefit of SRH may be limited for this population. Our results may help practitioners, whether in a clinical or research context, to understand the strengths and limitations of SRH for low-income populations from different age groups.

## Supplemental Information

10.7717/peerj.8876/supp-1 Supplemental Information 1 Study dataset.

10.7717/peerj.8876/supp-2 Supplemental Information 2 Results of the quantile regression models with interaction effect between the SRH and the age group (*N* = 571). SRH – Self-rated health. ^§^2 missing values. Model 1: Unadjusted. Model 2: Adjusted by age, education and family income. Model 3. Adjusted by age, education, family income, parity, age first birth and menopausal status. Model 4. Adjusted by age, education, family income, parity, age first birth, menopausal status, body mass index, physical activity and chronic conditions. Note: Higher values indicate better performance for grip strength and the balance tests, and worse performance for chair stands.

## Additional Information and Declarations

### Data Availability

The following information was supplied regarding data availability: Data are available in the Supplemental File.
