# Supplementary material for: Association between self-rated health and physical performance in middle-aged and older women from Northeast Brazil
Source: PeerJ. 2020 Apr 10;8:e8876. doi: 10.7717/peerj.8876 (PMC7153554; doi:10.7717/peerj.8876)
Supplement: Supplemental Information 2 — SRH – Self-rated health. §2 missing values. Model 1: Unadjusted. Model 2: Adjusted by age, education and family income. Model 3. Adjusted by age, education, family income, parity, age first birth and menopausal status. Model 4. Adjusted by age, education, family income, parity, age first birth, menopausal status, body mass index, physical activity and chronic conditions. Note: Higher values indicate better performance for grip strength and the balance tests, and worse performance for chair stands. [file peerj-08-8876-s002.docx]

**Supplemental file**. Results of the quantile regression models, with interaction effect between the SRH and the age group (N=571).

| **Performance measures** | **Parameters** | **Model 1** | **Model 2** | **Model 3** | **Model 4** |
| --- | --- | --- | --- | --- | --- |
|  |  | **b (95% CI)** | | | |
| **Grip strength (kgf)** | SRH: Good | 0.32  (-1.76 – 2.39) | 0.55  (-1.84 – 2.95) | 0.76  (-1.60 – 3.12) | -0.23  (-2.42 – 1.95) |
|  | *p value* | 0.825 | 0.508 | 0.681 | 0.896 |
|  | Age: Middle-age | 3.09  (1.71 – 4.47) | 2.83  (1.23 – 4.43) | 2.26  (0.76 – 3.77) | 2.34  (0.89 – 3.79) |
|  | *p value* | <0.001 | <0.001 | 0.001 | 0.001 |
|  | SRH: Good x Age: Middle-age | 1.53  (-0.82 – 3.88) | 1.48  (-1.17 – 4.13) | 1.04  (-1.65 – 3.72) | 1.82  (-0.67 – 4.31) |
|  | *p value* | 0.099 | 0.461 | 0.357 | 0.284 |
| **One legged balance - Eyes Open (s)**^(§)^ | SRH: Good | -0.57  (-8.39 – 7.26) | 0.06  (-7.05 – 7.18) | 0.12  (-8.02 – 8.26) | -0.32  (-7.47 – 6.82) |
|  | *p value* | 0.970 | 0.992 | 0.986 | 0.994 |
|  | Age: Middle-age | 13.68  (10.11 – 17.24) | 13.45  (9.80 – 17.10) | 12.87  (8.89 – 16.86) | 12.20  (8.13 – 16.27) |
|  | *p value* | <0.001 | <0.001 | <0.001 | <0.001 |
|  | SRH: Good x Age: Middle-age | 4.15  (-4.00 – 12.30) | 3.17  (-4.30 – 10.64) | 2.35  (-6.06 – 10.76) | 1.50  (-5.83 – 8.84) |
|  | *p value* | 0.338 | 0.448 | 0.456 | 0.779 |
| **One legged balance - Eyes Closed (s)**^(§)^ | SRH: Good | 0.92  (-0.15 – 1.99) | 1.02  (0.14 – 1.91) | 1.08  (0.05 – 2.12) | 0.81  (0.17 – 1.45) |
|  | *p value* | 0.069 | 0.035 | 0.040 | 0.032 |
|  | Age: Middle-age | 2.15  (1.37 – 2.94) | 1.97  (1.17 – 2.78) | 2.07  (1.09 – 3.05) | 2.19  (1.22 – 3.16) |
|  | *p value* | <0.001 | <0.001 | <0.001 | <0.001 |
|  | SRH: Good x Age: Middle-age | 0.61  (-1.24 – 2.47) | 0.52  (-1.19 – 2.24) | 0.27  (-1.44 – 1.99) | 0.94  (-0.45 – 2.34) |
|  | *p value* | 0.604 | 0.924 | 0.663 | 0.351 |
| **Chair Stands (s)**^(§)^ | SRH: Good | -1.33  (-2.55 – -0.10) | -1.33  (-2.70 – 0.04) | -2.01  (-3.21 – -0.80) | -1.41  (-2.60 – -0.22) |
|  | *p value* | 0.021 | 0.057 | 0.010 | 0.022 |
|  | Age: Middle-age | -1.80  (-2.70 – -0.90) | -1.74  (-2.70 – -0.78) | -1.85  (-2.76 – -0.94) | -1.65  -2.55 – -0.76) |
|  | *p value* | <0.001 | <0.001 | <0.001 | <0.001 |
|  | SRH: Good x Age: Middle-age | 0.79  (-0.51 – 2.09) | 0.82  (-0.64 – 2.27) | 1.57  (0.29 – 2.85) | 0.96  (-0.29 – 2.22) |
|  | *p value* | 0.211 | 0.194 | 0.041 | 0.097 |

SRH – Self-rated health. ^§^2 missing values.

Model 1: Unadjusted. Model 2: Adjusted by age, education and family income. Model 3. Adjusted by age, education, family income, parity, age first birth and menopausal status. Model 4. Adjusted by age, education, family income, parity, age first birth, menopausal status, body mass index, physical activity and chronic conditions.

Note: Higher values indicate better performance for grip strength and the balance tests, and worse performance for chair stands.
